# Laboratory evaluation of the efficacy and speed of kill of lotilaner (Credelio™) against *Ctenocephalides felis* on cats

**DOI:** 10.1186/s13071-018-2972-8

**Published:** 2018-07-13

**Authors:** Daniela Cavalleri, Martin Murphy, Wolfgang Seewald, Steve Nanchen

**Affiliations:** Elanco Animal Health, Mattenstrasse 24a, 4058 Basel, Switzerland

**Keywords:** Lotilaner, Credelio™, Cat, Flea, *Ctenocephalides felis*, Efficacy, Speed of kill, Safety

## Abstract

**Background:**

Lotilaner is approved for dogs as a chewable tablet formulation. It has separately been developed for oral administration in cats (Credelio™ chewable tablets for cats) to meet the need for an easy to use, safe and rapidly effective parasiticide and as an alternative to topical products. This paper describes two pivotal laboratory studies assessing the efficacy and speed of kill of lotilaner in cats against *Ctenocephalides felis* fleas following a single oral administration, at the minimum recommended dose rate of 6 mg/kg.

**Methods:**

Two GCP (Good Clinical Practice), blinded, randomized, negative-controlled, parallel-groups, laboratory studies were performed. In both studies, lotilaner was administered once, *per os*, at the minimum recommended dose of 6 mg/kg. Study 1 evaluated the efficacy of lotilaner tablets for cats against adult *C. felis* in experimentally infested cats, 24 h after treatment and after new weekly infestations, until day 35. Study 2 evaluated the speed of kill of lotilaner against *C. felis*, in cats, 8 and 12 h after treatment and after each subsequent weekly infestation, through day 35. In both studies, for each assessed time point, animals were randomized 1:1 to a lotilaner-treated or a contemporaneous negative control group of 8 cats each.

**Results:**

In both studies, the infestation in the control groups was adequate at all assessment times. In Study 1, efficacy at 24 h was 100% at all time points. In Study 2, efficacy was ≥ 97.4% at the 8 h and ≥ 98.6% at the 12 h time point, through one month. Lotilaner was well tolerated, with no product-related adverse events reported.

**Conclusions:**

Lotilaner administered orally to cats at the minimum recommended dose rate of 6 mg/kg was effective as early as 8 hours post-administration and at 8 hours after subsequent weekly infestations of adult *C. felis* for at least one month. The product was well-tolerated.

**Electronic supplementary material:**

The online version of this article (10.1186/s13071-018-2972-8) contains supplementary material, which is available to authorized users.

## Background

Although in the last decades, a number of new molecules have been approved and marketed for flea control in cats, with few exceptions, the most widely used route of administration has been the topical route. Topical flea control products have to be applied carefully directly to the pet’s skin and can potentially lead to pesticide exposure of all household members. It is also recognized that skin concentrations may unpredictably decline over the protection period following treatment, because of factors such as climate and water exposure [1]. Long-acting orally administered products overcome these shortcomings with the added advantage of potential to kill fleas more rapidly than topically applied flea control products, possibly because of the time needed for a topically applied product to homogeneously distribute from the site of application to the whole-body surface [2, 3]. Rapid killing of fleas is desirable to quickly remove the source of irritation from biting fleas, which can cause allergic dermatitis [4] and to break the flea life-cycle by preventing egg production.

Isoxazolines are a novel family of compounds that have a unique mode of action, inhibiting γ-aminobutyric acid (GABA)-gated chloride channels (GABACls) and glutamate-gated chloride channels (GluCls) leading to a progressive and irreversible paralysis of insects and acarines [5, 6]. They have been shown to be effective in treating ectoparasitic infestations in dogs [5–7]. The first approved isoxazolines (afoxolaner, fluralaner and sarolaner) are available as oral formulations for the treatment of flea and tick infestations in dogs.

The only isoxazolines approved for use in cats have been available for topical application, so far: fluralaner (Bravecto® spot-on solution for cats; Merck Animal Health, Madison, NJ, USA) and sarolaner in combination with selamectin (Stronghold® Plus spot-on solution for cats; Zoetis Belgium SA, Louvain-la-Neuve) [8, 9].

Lotilaner (Credelio™, Elanco, Greenfield, IN, USA) is the newest member of this class and is available as a flavoured chewable tablet formulation for dogs [10]. In order to fulfil the need for a rapidly effective, orally administered ectoparasiticide with activity against ticks and fleas in cats and kittens, Elanco has designed a small, vanilla and yeast flavoured, chewable lotilaner tablet, for oral administration to cats (Credelio™, chewable tablets for cats).

The efficacy and safety of Credelio™ against fleas and ticks in cats were validated in a number of pilot (undisclosed data) and pivotal laboratory studies [11]. In a pivotal tolerance study in 8-week-old kittens lotilaner tablets have been shown to be safe at doses up to 200 mg/kg for monthly treatment over 3 months and up to 130 mg/kg administered monthly for 8 months, respectively [12].

When administered with food, lotilaner is rapidly absorbed, with peak blood concentrations occurring within approximately 4 h, and has a half-life of approximately four weeks [13].

To provide confirmation of the efficacy of the minimum recommended dose of 6 mg/kg lotilaner and to evaluate the speed of kill throughout the month, two pivotal, GCP, assessor-blinded, randomised, negative-controlled studies, evaluated the efficacy and speed of kill of a single dose of lotilaner (Credelio™ chewable tablets for cats), against weekly experimental infestation with adult fleas, over 5 weeks.

## Methods

Two pivotal efficacy studies were conducted at an Elanco Animal Health laboratory in St-Aubin, Switzerland, in compliance with Swiss guidelines covering efficacy studies with ectoparasiticides in cats and dogs, VICH GL9 Good Clinical Practice Guidelines, EMEA/CVMP/EWP/005/2000-Rev.2, and European Parliament Directive 2001/82/EC [14–16].

### Design

Both studies were performed in compliance with GCP quality standards and were blinded, randomized, parallel-group and negative controlled.

Study 1 was a dose confirmation study with the objective of confirming the efficacy of lotilaner tablets for cats as close as possible to the minimum recommended dose rate of 6 mg/kg, against adult fleas (*Ctenocephalides felis*), in experimentally infested cats. Sixteen cats were randomized to two groups: one treatment group and one negative control group of eight cats each. Efficacy was evaluated 24 (± 1) h after treatment on day 0 (i.e. on day 1, 48 h after flea infestation) and 24 (± 1) h after each subsequent flea infestation (i.e. on days 8, 15, 22, 29 and 36).

The objective of Study 2 was to evaluate the speed of kill of lotilaner dosed orally once, on day 0, at the minimum recommended dose rate of 6 mg/kg against experimental infestations with adult fleas (*C. felis*) in cats. Thirty-two cats were randomized to four groups of eight animals each: two treatment groups (Groups 2 and 4) and two negative control groups (Groups 1 and 3). Efficacy was measured 8 and 12 h after treatment and 8 and 12 h after each subsequent weekly flea infestation, until the end of the study on day 35 (see Table 1 for details on cat groups and time points).

**Table 1:** Studies treatment groups and timing of flea counts

	Group	Treatment	Counting time	Infestation study days	Flea count days
Study 1	1 (*n* = 8)	Negative control	+24 h	-2, 7, 14, 21, 28, 35	1, 8, 15, 22, 29, 36
	2 (*n* = 8)	Lotilaner	+24 h	-2, 7, 14, 21, 28, 35	1, 8, 15, 22, 29, 36
Study 2	1 (*n* = 8)	Negative control	+8 h	-2, 7, 14, 21, 28, 35	0, 7, 14, 21, 28, 35
	2 (*n* = 8)	Lotilaner	+8 h	-2, 7, 14, 21, 28, 35	0, 7, 14, 21, 28, 35
	3 (*n* = 8)	Negative control	+12 h	-2, 6, 13, 20, 27, 34	0, 7, 14, 21, 28, 35
	4 (*n* = 8)	Lotilaner	+12 h	-2, 6, 13, 20, 27, 34	0, 7, 14, 21, 28, 35

A pre-dose infestation test was performed during the acclimatization phase in order to evaluate the flea retention rate measured at 24 (± 1) h post-infestation. This retention rate was used as a criterion for inclusion of the cats into the study and for randomization.

Body weight was recorded three times, twice during the acclimatization phase and then once at the end of the study. Full physical examinations were performed at the beginning of acclimatization and at the end of the study. General health observations were performed daily and additional clinical observations occurred at approximately 1 h, 6 h and 8 h post-treatment.

### Animals

In order to obtain eight cats suitable for inclusion in each study group, 60 healthy European domestic short hair cats of both sexes (24 cats in the first study and 36 cats in the second study) from the St-Aubin cat colony, with a minimum age of 12 months, with body weight that allowed for administration of lotilaner at a minimum dose of 6 mg/kg and as close as possible to this target dose rate, were selected for acclimatization. These cats had to be clinically healthy and with no pre-existing skin lesions or known high allergic reactions to insect bites. No other ectoparasiticide treatment had been provided to them for at least 8 weeks (6 months for isoxazolines) before the first infestation in these studies.

An infestation test (“pre-dose infestation test”) was performed during the acclimatization phase (14 days before start of the study) in order to evaluate the flea retention rate measured at 24 (± 1) h post-infestation. Cats having a flea retention rate of at least approximately 50% in the acclimatization phase were considered eligible for inclusion in the study. Any cats that developed important skin lesions or had an allergic reaction to insect bites after the pre-infestation test of the acclimatization phase and cats considered difficult to manipulate were excluded from the study. Following the acclimatization phase, 48 cats were randomized in the studies (16 cats in Study 1 and 32 in Study 2). After discussion between the investigator and the sponsor, three cats with flea retention rates slightly lower than 50% (47–49%) were included in Study 1. Similarly, one cat with a flea retention rate of 42% was included in Study 2. These deviations were minor and did not affect the study results. Any cats excluded from the study were returned to the research site cat colony.

Cats were housed in rooms, each containing two pens, with up to 8 cats per room and 4 cats per pen. In both studies, cats were housed in individual cages from infestations until the flea counts were completed. On some days during acclimatization, cats were also individually housed to be trained to consume their feeding portion in 30 min. Additional individual housing was applied on some acclimatization days for approximately 30 min or 1 h in Study 1 and 2, respectively, to train cats to wear an Elizabethan collar.

Commercially available cat food was provided once daily. To motivate cats to eat their whole food portion within 30 min before treatment, each cat received only half of their normal daily food portion the day prior to treatment (i.e. day -1). Cats were not force-fed. Fresh drinking water from the communal water plant was available *ad libitum*. Temperature and humidity were adequately controlled for the duration of the study and a few noted excursions were short and minor without any impact on the study outcome. During the study, cat toys and cat scratchers were available for animal welfare reasons.

### Randomisation and blinding

Randomization was carried out using SAS/STAT® software (ver. 9.2; SAS Institute Inc., Cary, NC, USA) per the site Standard Operating Procedure (SOP). Cats were ranked ordered from highest to lowest flea pre-dose counts and randomly allocated within blocks to treatment groups. Block sizes of two cats were used in Study 1 and of four cats were used in Study 2. When two or more cats had the same number of live fleas, the tie was broken randomly. Cats within each treatment group were randomly allocated to study rooms with each room containing an equal representation of treated and control cats of the same counting time point as applicable for the study. Blinding was accomplished by separation of function. In Study 1, study personnel responsible for general health observations, clinical observations, preparation of fleas, infestation with fleas, physical examination, safety data review, animal weighing, and/or animal maintenance were blinded throughout the study. The sponsor’s representative, monitor, quality assurance personnel, statistician, and dispenser and assistant were unblinded. In Study 2, blinding was guaranteed by a similar setting but the safety data review was performed by the unblinded sponsor’s representative.

### Flea infestation and counting

Except for the pre-dose infestation test, in the intervals between flea counting and next infestation, cats were combed for 8–16 min in order to remove any fleas remaining from the previous infestation.

A mixed flea strain of Swiss and Danish origin was used in both studies. This strain was confirmed as a *C. felis* strain based on morphological examination and confirmation that the morphological features were identical to those described by Zentko & Richman [17].

Flea infestations were performed per the site SOP: 100 (± 5) unfed adult *C. felis* fleas (both sexes and aged 17–21 days in Study 1 and 17–22 days in Study 2, were released on the back of the cats, from the neck to the central part of the spine. For each infestation period (from infestation until flea count was completed), cats wore an individual Elizabethan collar. The Elizabethan collar was removed prior to start combing the cats. Cats were not sedated for flea infestation.

For flea counts, cats were first combed for 10 min. If during the last 2 min, a minimum of three live fleas were found, the animal was combed for two additional min, for a maximum of 16 min combing time. During each flea count, all fleas were removed, and live fleas killed in 70% ethanol solution and/or in a freezer (for at least 24 h); all fleas were then disposed of in compliance with local requirements. No cat needed to be sedated for the flea counts.

In Study 1, fleas were classified as either live or dead. In Study 2, the additional category “moribund” was added. Fleas were considered live if they could maintain an upright posture and if they could actively move through hair removed from the animal during the combing procedure. A moribund flea was a flea that was laterally recumbent, could not normally move through hair or “right” itself when placed on a flat surface, but still had leg movement or twitching. A dead flea was a completely immobile flea.

### Treatment

Based on day -2 body weights, all cats in the treated groups received a single oral administration of lotilaner on day 0 at a minimum dose rate of 6 mg/kg and as close as possible to this target dose rate; the treatment was administered 30 ± 5 min following feeding. Two tablet strengths (12 mg and 48 mg) were available. The required number of tablets was placed over the tongue of the cats, in the back of the oral cavity followed by administration of 1–5 ml of water *via* a syringe to facilitate swallowing. This was followed by a check of each cat’s mouth to ensure that the full dose had been swallowed. Mock dosing was performed for cats in the negative control groups where the treatment administrator opened and massaged the cat’s mouth followed by administration of 1–5 ml of water *via* a syringe. In both studies, no animal receiving lotilaner spat out the tablet(s) or vomited within 30 to 45 min following dosing.

### Study assessments and statistical analyses

All statistical analyses were performed using SAS® (ver. 9.2.2, SAS Institute Inc., Cary, NC, USA). The experimental unit was the individual animal. Descriptive statistics (arithmetic and geometric mean, minimum, maximum, standard deviation, coefficient of variation and median) were calculated with respect to study groups separately for flea counts and body weights.

#### Efficacy

Efficacy was defined as the ability of lotilaner to reduce flea infestations on the cats at the specified time points (24 h in Study 1 and 8 h and 12 h in Study 2) after treatment following the infestation on day -2 and after each post-treatment infestation. Efficacy calculation was based on live flea counts; in Study 2, two different calculations were performed, in which moribund fleas were considered as either live or dead. Efficacy was determined based on the percent reduction in live adult flea counts in the treated group compared with the negative control (untreated) group for the same time point.

In Study 1, efficacy analyses were performed using the intent-to-treat data set, which included all randomized animals, i.e. animals that received lotilaner and the untreated animals. In Study 2, some cats could not be infested on some days for animal welfare reasons, due to reactions to flea bites, leading to smaller (but still sufficient) group sizes. On each day, data were present only for those animals which had been infested. Therefore, only these animals were included in the statistical analysis for efficacy (per protocol analysis).

Flea infestation was considered adequate at each time point after lotilaner administration if the arithmetic mean of the flea retention rate of the control animals was at least approximately 50%. There were separate calculations for each time point. Arithmetic and geometric means of the flea counts were calculated. Efficacy using geometric means was performed for information purposes only. Efficacy was calculated as follows:

$$ \%{\mathrm{Efficacy}}_{\mathrm{Arithmetic}/\mathrm{Geometric}}=100\times \left(\left(\mathrm{C}-\mathrm{T}\right)/\mathrm{C}\right) $$

where C is the arithmetic/geometric mean number of live fleas in the negative control group and T is the arithmetic/geometric mean number of live fleas in the treated group.

In order to avoid taking the log of zero, geometric means were calculated after adding 1 to all flea counts, and 1 was subtracted from the resulting geometric mean, if any of the flea counts were zero. Log-transformed flea counts were compared between groups in an analysis of variance (ANOVA). In Study 2, as the assumption of normal distribution of log-transformed flea counts was not valid, non-parametric methods were additionally applied to compare the treated and negative control groups (Mann-Whitney U-test).

Lotilaner was considered effective for the control of *C. felis* at a given time point if infestation was adequate, if there was a statistically significant difference between flea counts of the treated group and the untreated group (2-sided *P* < 0.05), and if the treated group had a calculated efficacy of ≥ 95% (live fleas) using the arithmetic means.

#### Safety

Adverse events were coded according to VeDDRA SOC and PT (Veterinary Dictionary for Drug Regulatory Activities, System Organ Class, Preferred Term). For each SOC and PT, the number of animals showing the respective sign was counted for untreated and treated animals, and the number of adverse events with the respective sign was calculated. Some of the adverse events that occurred during group housing could not be attributed to a specific cat but only to the set of four cats in the same pen. These adverse events were summarized separately, by pen rather than by cat. Full physical examinations were performed at the beginning of acclimatization and at the end of each study.

In Study 1, body weights were recorded on days -16, -9, -2 and 37. In Study 2, body weights were recorded on days -15, -9, -2 and 37 for Groups 1 and 2; and on days -16, -10, -3 and 36 for Groups 3 and 4. Body weight change was calculated as the change in weight from baseline (weight closest to dosing was used as baseline weight) to weight at the end of the study and was analysed in the first study using an analysis of covariance containing the model effects “Treatment Group” and “Baseline”. In Study 2, this was analysed using an analysis of variance (ANOVA) with the fixed effect of “Treatment Group” and the random effects of “Block” and “Room”.

French translation of the Abstract is available in Additional file 1.

## Results

In both studies, all cats in the lotilaner groups were dosed according to the protocol instructions. Since Credelio™ chewable tablets for cats are not divisible, animals were dosed with one or more full tablet(s), for the dose rate to be as close as possible to the target lotilaner dose of 6 mg/kg. The dose ranged from 6.2 to 8.3 mg/kg and from 6.5 to 8.1 mg/kg in Study 1 and 2, respectively. Cats in the negative control groups (untreated) were mock-dosed.

### Efficacy

#### Adequacy of infestation

In both studies, there was an adequate flea infestation rate of at least 50% in all the negative control groups at each of the time points. In Study 1, the arithmetic mean flea counts throughout the study were between 67.3–85.4% in the control group. In Study 2, when moribund fleas were considered live, they were between 79.6–85.6% in the 8 h control group and between 69.6–86.9% in the 12 h control group.

#### Efficacy percentage

**Fig. 1:**
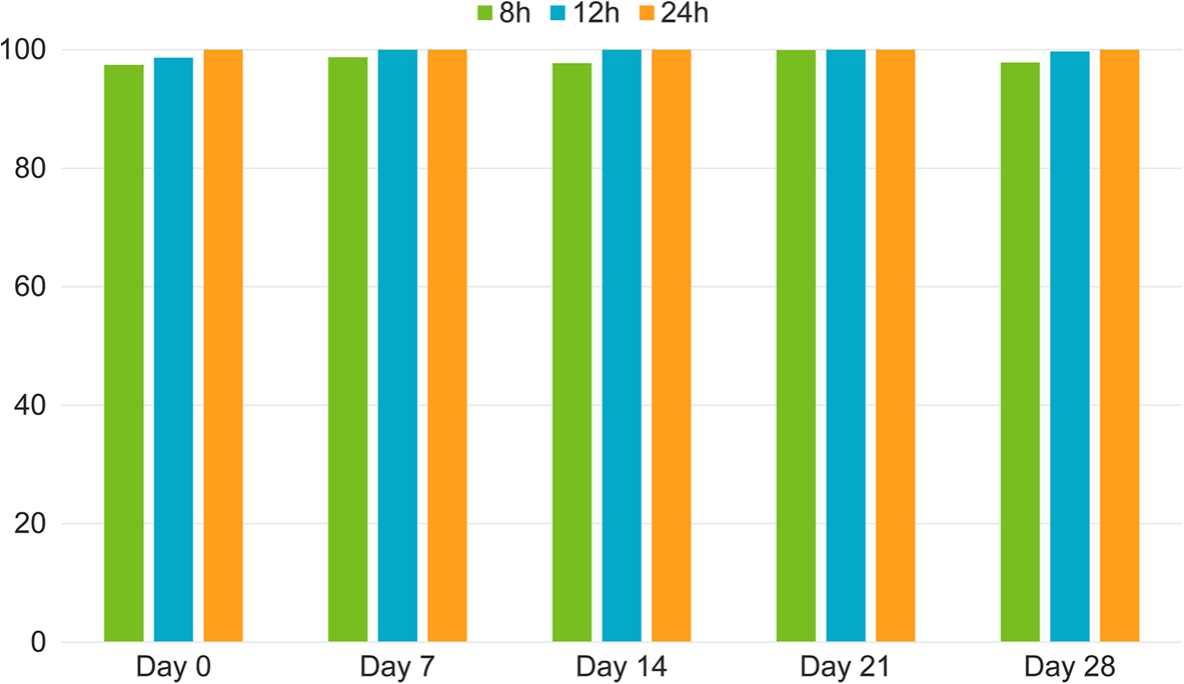
Percentage of efficacy based on arithmetic means on Day 0 at 8, 12 and 24h post-treatment; and on Day 7, 14, 21 and 28 at 8, 12 and 24 h post-infestation. (*P* ≤ 0.0008).

Study 1: Arithmetic and geometric mean flea numbers in the lotilaner group were 0 at all time points, corresponding to a calculated efficacy of 100% on all assessment days. Differences in flea counts between treatment groups were significant at all time points (Table 2 and Fig. 1).

**Table 2:** Mean flea counts in control and lotilaner-treated animals and % efficacy at 24 hours post-treatment and 24 hours after weekly infestations (Study 1)

		Day of infestation and flea count					
		0	7	14	21	28	35
Control (n = 8)	Arithmetic mean ± SD	67.3 ± 14.3	85.4 ± 8.7	79.6 ± 10.7	75.1 ± 12.2	71.4 ± 10.3	84.4 ± 11.4
	Geometric mean ± SD	65.8 ± 15.1	85.0 ± 8.8	79.0 ± 10.8	74.2 ± 12.9	70.8 ± 10.0	83.7 ± 12.2
Lotilaner (n = 8)	Arithmetic mean ± SD	0.0 ± 0.0	0.0 ± 0.0	0.0 ± 0.0	0.0 ± 0.0	0.0 ± 0.0	0.0 ± 0.0
	Efficacy (%)	100	100	100	100	100	100
	Geometric mean ± SD	0.0 ± 0.0	0.0 ± 0.0	0.0 ± 0.0	0.0 ± 0.0	0.0 ± 0.0	0.0 ± 0.0
	Efficacy (%)	100	100	100	100	100	100
Comparison		*t*_(13)_ = 57.1, *P* < 0.0001	*t*_(13)_ = 136.9, *P* < 0.0001	*t*_(13)_ = 99.9, *P* < 0.0001	*t*_(13)_ = 71.7, *P* < 0.0001	*t*_(13)_ = 86.8, *P* < 0.0001	*t*_(13)_ = 87.3, *P* < 0.0001

Study 2: When based on arithmetic mean and with moribund fleas considered as live, at the 8 h time point, the efficacy of lotilaner was 97.4% after treatment on day 0 and ≥ 97.7% after new subsequent infestations until the end of the month; on day 35 it was 91.6% (Table 3 and Fig. 1).

Efficacy at 8 h post-treatment and post re-infestations based on geometric means was similar and ranged from 98.3 to 99.9% from day 0 to day 28 and 94.5% on day 35. Mean flea numbers and corresponding efficacy percentages, along with comparison *P*-values by time point are shown in Table 3.

**Table 3:** Mean flea counts in control and lotilaner-treated animals and % efficacy at 8 hours post-treatment and 8 hours after weekly infestations (Study 2)

		Number of animals, day of infestation and flea count											
		*n*	0	*n*	7	*n*	14	*n*	21	*n*	28	*n*	35
Control	Arithmetic mean ± SD	8	82.4 ± 8.1	7	79.6 ± 12.7	7	82.7 ± 15.6	6	83.7 ± 7.7	7	85.6 ± 13.5	7	81.6 ± 9.7
	Geometric mean		82.0		78.7		81.4		83.4		84.7		81.1
Lotilaner	Arithmetic mean ± SD	8	2.1 ± 3.6	8	1.0 ± 1.4	8	1.9 ± 0.1	8	0.1 ± 0.4	8	1.9 ± 1.5	8	6.9 ± 7.1
	Efficacy (%)		97.4		98.7		97.7		99.9		97.8		91.6
	Geometric mean		1.0		0.7		1.7		0.1		1.5		4.5
	Efficacy (%)		98.8		99.2		97.9		99.9		98.3		94.5
Comparison			*t*_(6)_ = 11.3, *P* < 0.0001		*t*_(5)_ =17.9, *P* < 0.0001		*t*_(5)_ = 25.0, *P* < 0.0001		*t*_(4)_ = 42.7, *P* < 0.0001		*t*_(5)_ = 15.4, *P* < 0.0001		*t*_(5)_ = 7.3, *P* = 0.0008

*Abbreviation*: *SD* standard deviation

In the same study, at the 12 h time point, when moribund fleas were considered as live, efficacy ranged from 98.6 to 100% when based on arithmetic mean flea counts (Table 4 and Fig. 1), and 99.3 to 100% when geometric means were applied (Table 4).

**Table 4:** Mean flea counts in control and lotilaner-treated animals and % efficacy at 12 hours post-treatment and 12 hours after weekly infestations (Study 2)

		Number of animals, day of infestation and flea count											
		*n*	0	*n*	7	*n*	14	*n*	21	*n*	28	*n*	35
Control	Arithmetic mean ± SD	8	69.6 ± 14.4	8	86.9 ± 14.5	8	76.6 ± 11.4	8	85.0 ± 9.7	8	83.1 ± 11.1	8	84.4 ± 5.0
	Geometric mean		68.3		85.7		75.9		84.5		82.4		84.2
Lotilaner	Arithmetic mean ± SD	8	1.0 ± 21	7	0.0 ± 0.0	7	0.0 ± 0.0	7	0.0 ± 0.0	7	0.3 ± 0.5	7	1.1 ± 2.3
	Efficacy (%)		98.6		100		100		100		99.7		98.6
	Geometric mean		0.5		0		0		0		0.2		0.5
	Efficacy (%)		99.3		100		100		100		99.7		99.4
Comparison			*t*_(6)_ = 15.5, *P* < 0.0001		*t*_(5)_ = 66.3, *P* < 0.0001		*t*_(5)_ = 74.7, *P* < 0.0001		*t*_(5)_ = 280.6, *P* < 0.0001		*t*_(5)_ = 39.0, *P* < 0.0001		*t*_(5)_ = 15.0, *P* < 0.0001

*Abbreviation*: *SD* standard deviation

For both the 8 h and 12 h assessment time points, differences in flea counts between treatment groups were significant on all study days.

When only live flea counts were used for efficacy calculation, efficacy percentages for the 8 h assessment, increased to 96.2–100% and 97.6–100% (based on arithmetic and geometric means, respectively), while for the 12 h time point the corresponding ranges were 99.0–100% and 99.6–100%.

### Safety

All included cats remained in both studies until the end of the animal phase. In Study 1, all adverse events (AEs) reported (4 in treated cats and 1 in an untreated cat) were considered as unrelated to the product: 4 of them were skin-related and likely due to the repeated flea infestation; one cat in the treated group had an episode of vomiting on day 22. All cases were reported as mild, there was no need to administer concomitant treatment and all cats made a full recovery.

In Study 2, nineteen skin related adverse events were reported in the untreated groups and fifteen in the treated ones. All were attributed to the repeated flea infestations.

A number of mild gastrointestinal observations (loose stool, vomiting of hairball, and sporadic cases of blood in faeces) were also reported: 64 in the untreated and 43 in the lotilaner-treated groups. The exact number of cats affected by these adverse events could not be computed due to the fact that most observations occurred during group housing and were attributed to all animals in the same pen. There were two serious adverse events (one hotspot and one skin abscess) that occurred in untreated animals which needed treatment with antibiotics and corticosteroids.

The ANOVA model revealed no statistically significant differences in body weight change (from baseline to end of the study) between the treated and untreated groups.

## Discussion

These two studies demonstrated the efficacy of lotilaner (Credelio™ chewable tablets for cats) against experimental flea infestations as soon as 8 hours post-administration and after subsequent infestations, for one month. Since Credelio™ chewable tablets for cats are not divisible, animals were dosed with full tablets. The importance of a highly efficacious and fast killing flea-product is linked to the fact that, after infesting the host, fleas start feeding almost immediately and flea egg production begins within 24–36 h after the first blood meal has been taken [18, 19]. The main objectives of a flea control programme should be to treat flea infestations fast enough to limit the exposure of the animal to the flea salivary antigens and to possible pathogens, thus reducing the risk of FAD (flea allergy dermatitis), and of vector-borne diseases. In addition, the use of a fast flea-killing product allows the prevention of flea egg production and contributes to the depletion of the parasite stages in the cat’s environment.

The results of the two studies show that efficacy, starting 8 hours post-treatment and weekly re-infestations increases with time reaching 100% at 24 hours. In Study 2, two different approaches for the calculation of efficacy were applied, with moribund fleas being considered as live or dead. At the time the study was performed, the approach of the EU and USA regulators concerning the classification of the moribund fleas seemed to be changing, and the consensus position adopted was to consider these fleas as live, therefore relevant for efficacy calculation. For this reason, the primary efficacy objective in Study 2 included moribund fleas in the calculation. Still, the authors considered the investigation of efficacy with moribund fleas disregarded, as possibly providing a deeper understanding of the killing activity of the product.

At the 8 h time point, higher efficacy percentages were obtained when moribund fleas were excluded from the calculation, while at 12 h, the difference between the two calculations was less prominent. This comparison, together with the observation that, in the control groups, mean live flea counts were identical when moribund fleas were included in the counts or disregarded, suggest that moribund fleas had been affected by lotilaner but the early 8 h time point had not allowed yet for their complete death.

The fast and sustained speed of kill of Credelio™ is consistent with pharmacokinetic data, showing that, following oral administration in fed cats, lotilaner is readily absorbed, with peak blood concentrations reached within four hours and a terminal half-life of about 4 weeks [13].

The duration of efficacy of Credelio™ until the end of the month, is a guarantee for both cat owners and veterinarians that new flea infestations will be knocked-down very rapidly, helping preventing the onset or the re-appearance of the signs of FAD in cats with a confirmed diagnosis of FAD. By quickly killing newly infesting fleas before they can lay eggs, monthly treatments will break the flea life-cycle in the cat environment and will prevent environmental flea contamination eliminating the need for additional environmental control products, e.g. insect growth regulators. The main focus of these studies was the efficacy evaluation of Credelio™; therefore neither haematological nor biochemical analyses were performed. Data from the target animal safety study [12], in which the product was administered orally every 4 weeks for 8 months, at doses up to 130 mg/kg lotilaner, in healthy kittens, 8-week-old at study start, showed no clinically relevant treatment-related effects on clinical pathology parameters and no treatment-related effects on food consumption, ophthalmoscopic, physical/neurological and organ macroscopic and microscopic examinations.

These observations, together with the absence of product-related adverse events in the efficacy studies presented here, and the overall higher rate of skin and gastrointestinal observations in control animals, confirm that Credelio™ is well tolerated in cats and kittens. The clinical safety and the efficacy of the product against fleas and clinical signs of FAD, were further confirmed in an European, well controlled, randomized pivotal field study [20], assessing the efficacy of Credelio™ when administered to privately-owned cats, naturally infested with fleas, according to the proposed commercial label.

In this study, the efficacy and safety of lotilaner were compared to those of a fipronil/(S)-methoprene spot-on. The overall flea reduction for the study period was 97.7% in cats treated with lotilaner compared with a reduction of 47.4% for cats treated with fipronil/(S)-methoprene. Credelio™ superiority to the control product was demonstrated at all time-points and for the entire study period (*P* < 0.0001). At every post-administration evaluation, at least 81% of lotilaner-treated cats were flea-free as opposed to 25% in the fipronil/(S)-methoprene group. Credelio™ improved or eliminated clinical signs of FAD, including pruritus.

In a similar European field study, focussing on safety and efficacy against ticks, lotilaner was compared to a fipronil spot-on. The mean percent efficacy over all post-enrolment visits was 99.6% and 96.4% (lotilaner and fipronil group, respectively) (*P* < 0.0001). Lotilaner was superior to fipronil for efficacy averaged over all time points (*P* < 0.0001) and on individual assessment days (day 14 to 70, *P* < 0.0394); it was non-inferior to fipronil on the other days.

In the two field studies, Credelio™ was well tolerated and Fisher’s exact test showed that the number of cats affected by adverse events was not significantly different between the two treatment groups for each of the signs.

Historically, the availability of products that treat cats for both ticks and fleas has been quite limited. Fipronil has been available as a topical for many years in products like Frontline® Combo and Broadline® (Merial) [21, 22]. A collar combining imidacloprid and flumethrin (Seresto®) is also currently available in many markets [23]. More recently, Bravecto® spot-on solution (fluralaner; MSD/Merck) and Stronghold® Plus (selamectin/sarolaner; Zoetis), both of which contain topical isoxazolines, were registered for cats [8, 9]. Credelio™ (lotilaner) represents both the first oral tick and flea product as well as the first oral isoxazoline registered for cats.

## Conclusions

Lotilaner administered orally at a minimum dose rate of 6 mg/kg to cats was effective as early as 8 hours post-administration and subsequent new re-infestations for at least one month, against experimental infestations of adult *C. felis*. Eight hours after dosing and after new infestations, efficacy was at least 97.4%, increasing to 98.6% at 12 hours and reaching 100% at 24 hours, through one-month post-administration. By quickly killing pre-existing and newly infesting fleas, lotilaner has the potential to contribute to the reduction of flea-borne pathogens transmission, the development and re-occurrence of FAD and breaking the flea life-cycle in the cat environment. Credelio™ was well-tolerated with no product-related adverse events recorded.

## Additional file

Additional file 1: French translation of the Abstract. (PDF 64 kb)

## Abbreviations

AE: Adverse event
ANOVA: Analysis of variance
EU: European Union
FAD: Flea allergy dermatitis
GCP: Good clinical practice
PT: Preferred term
SD: Standard deviation
SOC: System organ class
SOP: Standard operating procedure
