# Supplementary material for: Laboratory evaluation of the efficacy and speed of kill of lotilaner (Credelio™) against Ctenocephalides felis on cats
Source: Parasit Vectors. 2018 Jul 13;11:408. doi: 10.1186/s13071-018-2972-8 (PMC6043949; doi:10.1186/s13071-018-2972-8)
Supplement: Supplementary file 1 — French translation of the Abstract. (PDF 64 kb) [file 13071_2018_2972_MOESM1_ESM.pdf]

Évaluation en laboratoire de l'efficacité et de la rapidité d'action du lotilaner (Credelio™) contre les puces *Ctenocephalides felis* chez le chat

Daniela Cavalleri[1*], Martin Murphy[1], Wolfgang Seewald[1] et Steve Nanchen[1]

[1]Elanco Animal Health, Mattenstrasse 24a, CH-4058, Bâle, Suisse.

Correspondance : cavalleri_daniela_a@elanco.com

Adresses électroniques :
Daniela Cavalleri : cavalleri_daniela_a@elanco.com
Martin Murphy : murphy_martin_gerard@elanco.com
Wolfgang Seewald : seewald_wolfgang@elanco.com
Steve Nanchen : nanchen_steve@elanco.com

Résumé

Contexte : Le lotilaner est autorisé chez le chien sous forme de comprimés à croquer. Il a été développé en parallèle en vue d'une administration orale chez le chat (comprimés à croquer pour chats Credelio™) afin de proposer un antiparasitaire pratique, sans danger et efficace rapidement, qui offre une alternative aux produits topiques. Cette publication décrit deux études pivots de laboratoire dont l'objectif était d'évaluer l'efficacité et la rapidité d'action du lotilaner contre les puces de l'espèce *Ctenocephalides felis* chez le chat, suite à une seule administration par voie orale, à la dose minimale recommandée de 6 mg/kg.

Méthodes : Deux études de laboratoire randomisées, réalisées en aveugle, en groupes parallèles et contre témoins ont été menées conformément aux principes de BPC (bonnes pratiques cliniques). Dans chacune d'elles, le lotilaner a été administré une seule fois *per os*, à la dose minimale recommandée de 6 mg/kg. Sur des chats ayant fait l'objet d'une infestation expérimentale, l'étude 1 a évalué l'efficacité des comprimés pour chats à base de lotilaner contre les puces *C. felis* adultes, 24 heures après le traitement puis après chaque nouvelle infestation hebdomadaire jusqu'à J35. L'étude 2 a quant à elle évalué la rapidité d'action du

lotilaner contre les puces *C. felis* sur des chats, 8 à 12 heures après le traitement puis après chaque nouvelle infestation hebdomadaire, jusqu'à J35. Dans les deux études et pour chaque évaluation, les animaux ont été répartis aléatoirement selon un rapport 1/1 entre un groupe traité par lotilaner et un groupe témoin parallèle de 8 chats chacun.

Résultats : L'infestation était suffisante dans les groupes témoins des deux études lors de toutes les évaluations réalisées. Dans l'étude 1, l'efficacité à 24 h était de 100 % lors de toutes les évaluations réalisées. Dans l'étude 2, l'efficacité était ≥ 97,4 % à 8 h et ≥ 98,6 % à 12 h pendant un mois. Le lotilaner a été bien toléré, et aucun événement indésirable imputable au produit n'a été signalé.

Conclusions : Le lotilaner administré par voie orale à des chats à la dose minimale recommandée de 6 mg/kg, s'est avéré efficace dès 8 h post-administration et 8 h après les infestations hebdomadaires suivantes par des puces *C. felis* adultes et ce pendant au moins un mois. Le produit a été bien toléré.

Mots clés : Lotilaner, Credelio™, chat, puce, *Ctenocephalides felis*, efficacité, rapidité d'action, innocuité.
